# Influence of Percutaneous Drainage Surgery and the Interval to Perform Laparoscopic Cholecystectomy on Acute Cholecystitis through Genetic Algorithm-Based Contrast-Enhanced Ultrasound Imaging

**DOI:** 10.1155/2022/3602811

**Published:** 2022-07-30

**Authors:** Qiaoying Li, Rong Cheng, Xiao Gao, Limin Zhu

**Affiliations:** ^1^Department of Ultrasound, Tangdu Hospital, Xian 710000, Shaanxi, China; ^2^Department of Ultrasound, No. 215 Hospital of Shaanxi Nuclear Industry, Xianyang 712000, Shaanxi, China

## Abstract

To discuss the optimal interval time between genetic algorithm-based ultrasound imaging-guided percutaneous drainage surgery (PTGD) and laparoscopic cholecystectomy (LC), 64 cholecystitis patients were selected as the research objects and evenly divided into experimental group (intelligent algorithm was adopted to recognize patients' ultrasonic images) and control group (professional doctors carried out diagnosis). 92 acute cholecystitis patients undergoing PTGD were divided into three groups. 30 out of the 92 patients received LC within 2 months and were defined as the early group. 32 were performed with LC within 2 to 4 months and were defined as the metaphase group. 28 underwent LC over 4 months and were defined as the late-stage group. The average operation time, the transition from LC to laparotomy, the average postoperative hospital stay, and the incidence of complications of the three groups were compared. The results revealed that the comparison of the diagnostic accuracy and comprehensive effectiveness between experimental group and control group demonstrated that the differences were statistically significant (*P* < 0.05). When the optimal interval of implementing LC after PTGD was realized, the corresponding values of the early group were 88.5 minutes, 16.67%, 8.13 days, and 13.75%. Those of the metaphase group were 49.91 minutes, 3.13%, 4.97 days, and 9.52%. Those of the late stage group were 68.78 minutes, 10.71%, 7.09 days, and 11.96%. To sum up, the diagnostic accuracy and comprehensive effectiveness of intelligent algorithm were higher than those of conventional ultrasound, and the optimal interval time of implementing LC after PTGD was 2 to 4 months.

## 1. Introduction

Acute cholecystitis (AC) is a common acute gastrointestinal disease arising from bile duct obstruction and bacterial infection. It has a rapid onset and requires surgical treatment in severe cases. The typical symptom is fever [1], and patients usually suffer from intense pain in the upper right abdomen [2]. It is generally treated by the surgery. If treated by medication alone, it is easy to relapse and worsen. 10% patients have serious complications; the mortality rate within one year is 3%, and the recurrence rate within one year is 30% [3]. As for the surgical treatment, the mortality rate is 0.5%, and the best time for surgery is within one week of onset [4]. Laparoscopic cholecystectomy (LC) is the main surgical method. Early surgery is defined as the surgery performed one week to two months after the onset [5], and the delayed surgery is one performed four weeks after the onset [6]. Early surgery is safer than delayed surgery, with a lower mortality rate and less complications [7]. However, for patients with multiple diseases, it is dangerous to have LC, which will negatively affect their life and health [8]. Percutaneous transhepatic gallbladder drainage (PTGD) is guided by X-ray or B-ultrasound [9]. Specifically, a special puncture needle is used to penetrate the intrahepatic bile duct through the skin, and then the contrast agent is directly injected into the bile duct for rapid development, and at the same time, biliary drainage is performed [10]. After PTGD, the bile is usually dark green in 1 to 2 days and then gradually becomes clear yellow or yellow-green. PTGD is easy to operate and can quickly alleviate the clinical symptoms of AC [11, 12].

Contrast-enhanced ultrasound is a technique that uses a contrast agent to enhance the scattered echo, thereby improving the resolution, sensitivity, and specificity of diagnosis [13]. With the improvement of instruments, it is possible to more clearly observe the blood perfusion of diseased tissue, and thus contrast-enhanced ultrasound is full of promises [14]. In general, contrast-enhanced acoustics are superior to conventional ultrasound and computed tomography (CT), and they have the same sensitivity as CT [15]. Compared with CT and MRI, contrast-enhanced ultrasound has advantages over safety, allergic reactions, and the costs. Intelligent algorithm refers to some algorithms or theories which can solve complex engineering problems [16]. There are many intelligent recognition algorithms. For example, the intelligent algorithms for processing and optimizing ultrasound images include genetic algorithm (GA), ant colony algorithm, simulated annealing algorithm, and particle swarm algorithm. GA is a computational model that simulates the natural selection and genetic evolution processes of biological evolution theory [17]. It searches for the optimal solution by simulating the natural evolution process. It can directly operate on structural objects and is not limited by function continuity. It is characterized by the parallelism and the ability of global optimization [18]. As for the solving steps of GA, first, the mapping of the solution space of the problem to the chromosome coding space is identified [19]; then, the population is initialized under certain restricted conditions and each chromosome in the population is decoded into a function form to select the best reproduction [21]. In this study, the contrast-enhanced ultrasound images are processed by the GA algorithm, and then the optimal interval for implementing LC after PTGD is investigated, expected to provide a theoretical basis for the clinical treatment of AC.

## 2. Materials and Methods

### 2.1. Research Subjects and the Grouping

To analyze the effect of GA on contrast-enhanced ultrasound images, a total of 64 patients diagnosed with cholecystitis in the hospital from March 2019 to May 2021 were selected as research subjects, including 50 patients with AC. Of the 64 patients, there were 31 male patients and 33 female patients, aged between 23 and 67. Patients who withdrew and were transferred to other hospitals were excluded. The patients were randomly divided into two groups with 32 cases in each group. The GA group used GA algorithm to process the ultrasound images of patients with cholecystitis to determine the severity of cholecystitis, so as to more accurately determine whether PTGD and LC were required. The control group had a professional doctor to directly diagnose the patient's contrast-enhanced ultrasound images. This study has been approved by ethics committee of hospital. The patient and his family members had signed an informed consent form.

Inclusion criteria were as follows: (I) patients diagnosed with cholecystitis; (II) patients who had not received other medication treatment; (III) patients who had not undergone the gallbladder surgery; (IV) patients with no history of cholecystitis; and (V) patients aged between 18–80 years old.

Exclusion criteria were as follows: (I) patients with contraindications to contrast-enhanced ultrasound; (II) patients with other system diseases and other organ dysfunction; (III) patients with incomplete clinical data and information; (IV) patients who did not cooperate with doctors throughout the process; and (V) patients who were pregnant, breastfeeding or had allergies.

### 2.2. Genetic Algorithm (GA)

**Figure 1:**
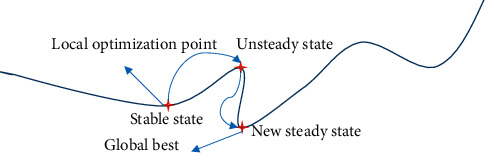
Flowchart of the GA algorithm.

GA is an iterative adaptive probability search method based on natural selection and natural genetics. It simulates the biological evolution process in nature and embodies the optimization idea of natural selection for survival of the fittest. It has good robustness and adaptability and can find global optimization solutions. From local optimization to global optimization, it searches for the best through mutation methods such as gene mutation, as shown in Figure 1.

### 2.3. Contrast-Enhanced Ultrasound

Color Doppler ultrasound system was used. Contrast-enhanced ultrasound is a new type of contrast technology that can clearly and accurately visualize the shape of the target part. The contrast agent was used to enhance the backscattered echo and significantly improve the resolution, sensitivity, and specificity of diagnosis. Contrast-enhanced ultrasound technology is often used in the diagnosis and identification of liver tumors. During the examination, a routine ultrasound examination is performed first to understand the location, size, shape, boundary, and echo characteristics of the lesion. Then, color Doppler and spectral Doppler are performed to detect the blood flow characteristics of the lesion. Later, the ultrasound contrast agent is injected intravenously for contrast-enhanced ultrasound examination. The entire process takes about 15–30 minutes. Patients generally do not need special preparation before the examination. To detect the gallbladder and pancreatic lesions, the patient needed to fast for more than 8 hours before the ultrasound examination. Also, appropriate breath training was required. After the examination, the patient should be observed for at least 15 minutes. Any discomfort such as flustering and chest tightness should be reported to the doctor in time.

### 2.4. Surgical Methods

The PTGD operation was guided under the ultrasound contrast based on GA. The patient was in a prone position to raise the right torso. Under the guidance of B-ultrasound, the puncture needle drew the bile and injected it to the catheter sheath along the needle core. Then, the needle core was removed, and the catheter was fixed and connected to the drainage bag. After general anesthesia during LC operation, the patient slept on his right side with a head-high and foot-low position. The umbilicus was punctured to establish the pneumoperitoneum, and another 3 holes were selected in the upper middle abdomen to insert the instrument to expose the gallbladder, free adhesions around the gallbladder and the triangle area of the gallbladder. When it was confirmed that the neck duct of the gallbladder joined the common bile duct, the distal end of the neck duct of the gallbladder was clamped, and a small cut was made at the proximal end to eliminate residual stones by pushing from the confluence of the neck duct of the gallbladder to the distal end until clear bile appeared. Again, the proximal end of the cystic duct was clamped, the gallbladder blood vessels were separated. During LC, if there are severe gallbladder delta adhesion, frozen appearance, suspected adhesion of biliary tract or surrounding organs, such as duodenum and colon, the laparotomy is required.

### 2.5. Evaluation Index

The ultrasound contrast images were processed by GA algorithm to diagnose AC, and the diagnostic results were compared with the diagnostic results by the doctor. Assume that AC is a positive case, and nonacute is a negative case. True positive (TP) means that the positive case is predicted as positive. False positive (FP) means that the negative case is predicted as positive. False negative (FN) means that the positive case is predicted as negative. True negative (TN) means that the negative case is predicted as negative. In this study, the accuracy rate is used to indicate the proportion of patients who are correctly predicted. The calculation method is shown in the following (1) and abbreviated as A; precision means the proportion of correctly predicted positive cases in all predicted positive cases, and the specific calculation method is shown in (2) and abbreviated as P; recall is the ratio at which a positive case is predicted, and the specific calculation method is shown in (3) and abbreviated as *R*.

(1) $$\begin{array}{c} A=\frac{\text{TN}+\text{TP}}{\text{TN}+\text{TP}+\text{FP}+\text{FN}}, \end{array}$$

(2) $$\begin{array}{c} P=\frac{\text{TP}}{\text{TP}+\text{FP}}, \end{array}$$

(3) $$\begin{array}{c} R=\frac{\text{TP}}{\text{TP}+\text{FN}}. \end{array}$$

The two indicators of *R* and P are often inconsistent. To take both of them into consideration, F-measure is often used, which is the weighted harmonic average of P and *R*, as shown in equation (4). When *α* = 1, as shown in equation (5), a higher F1 value indicates higher effectiveness and feasibility of the algorithm.

(4) $$\begin{array}{c} F=\frac{\left(a^{2}+1\right)P*R}{a^{2}\left(P+R\right)}, \end{array}$$

(5) $$\begin{array}{c} F1=\frac{2P*R}{P+R}. \end{array}$$

Different interval groups were compared for the surgical time of LC, the transition from LC to laparotomy, postoperative hospital stay, and complications, so as to analyze the optimal interval.

### 2.6. Statistical Methods

The data processing of this study used SPSS version 19.0 statistical software. The measurement data were expressed as mean ± standard deviation ($\bar{x}$ ± *s*), and the count data were expressed as percentage (%). Pairwise comparison used analysis of variance. *P* < 0.05 indicated a statistically significant difference.

## 3. Results

### 3.1. Comparison between Contrast-Enhanced Ultrasound and Ordinary Two-Dimensional Ultrasound

**Figure 2:**
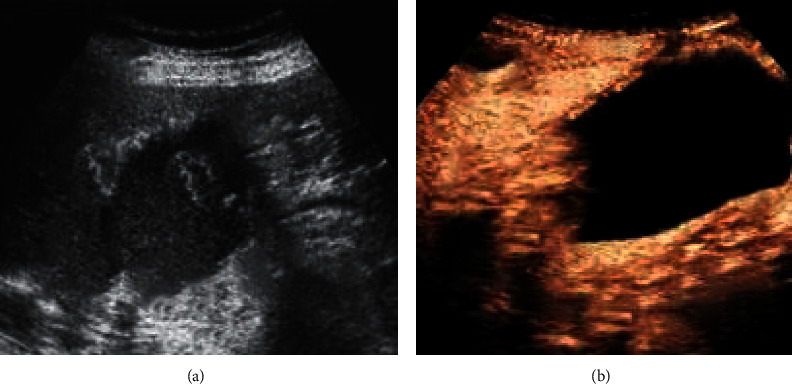
Contrast ultrasound image: (a) two-dimensional ultrasound showing thickening of the gallbladder wall, multiple stones, and linear echo in the cyst cavity, which was considered the intima shedding; (b) contrast ultrasound showing discontinuous enhancement of the gallbladder wall, with focal defects, and the falling intima within the cyst cavity was not reinforced.

The following is ultrasound images of three patients. Based on the time to coagulate the contrast agent of different tissue, the degree of adhesion of the tissue around the gallbladder and the integrity of the gallbladder wall were identified. As shown in Figure 2, a 67-year-old woman complained of pain in her right upper abdomen. She was diagnosed with AC and her white blood cell count was normal.

**Figure 3:**
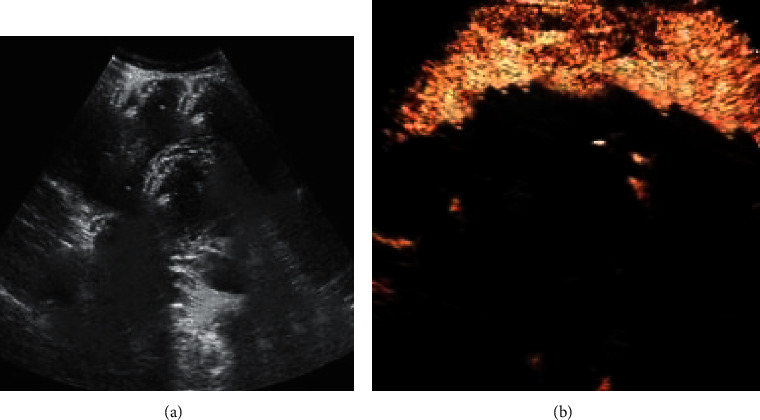
Contrast ultrasound image: (a) two-dimensional ultrasound image; (b) contrast-enhanced ultrasound.

Figure 3 shows images of a 62-year-old woman complaining of upper abdominal pain, diagnosed as having AC.  Figure 3(a) shows multiple gallstones and negative Murphy sign. 25 seconds after the injection of contrast agent, there is no enhancement in the gallbladder wall dilated area in Figure 3(b), which was caused by ischemia. The gallbladder wall showed segmental enhancement.

**Figure 4:**
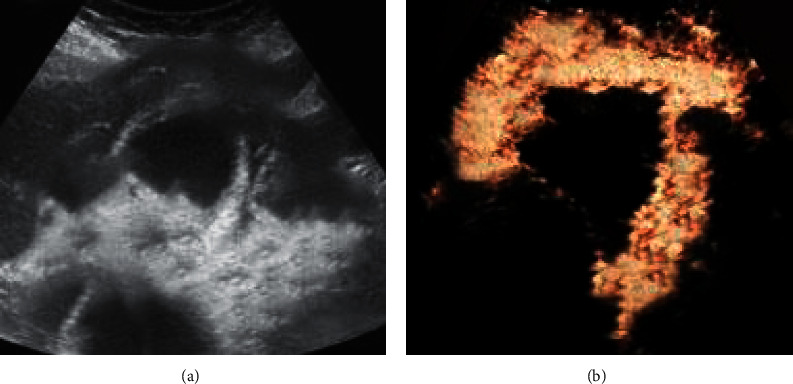
Contrast ultrasound image: (a) two-dimensional ultrasound image of the cross section of the gallbladder; (b) contrast-enhanced ultrasound.

Figure 4 shows images of an 86-year-old woman who complained of pain and leukocytosis in the right upper quadrant and was diagnosed as having acute gangrenous cholecystitis.  Figure 4(a) shows the thickening of the gallbladder wall, multiple gallbladder stones with acoustic shadows, secondary duodenal wall edema, and positive Murphy sign.  Figure 4(b) shows irregular gallbladder wall enhancement, and the arrow indicates a discontinuous area, suggesting gallbladder wall ischemia.

### 3.2. PTGD under the Guidance of Contrast-Enhanced Ultrasound

**Figure 5:**
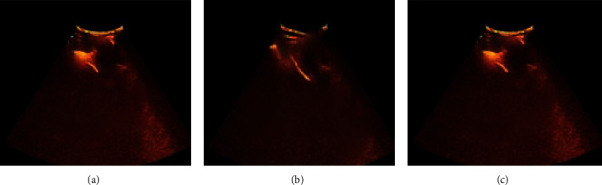
Contrast-enhanced ultrasound of drainage tube after puncture: (a–c) the contrast-enhanced ultrasound images of three patients after the PTGD.

Figure 5 shows the ultrasound image of the gallbladder of three patients after PTGD. The contrast agent was fully distributed in the gallbladder cavity so that the patency of the drainage tube and its position in the gallbladder can be monitored in real time.

### 3.3. Performance of GA Contrast-Enhanced Ultrasound

**Figure 6:**
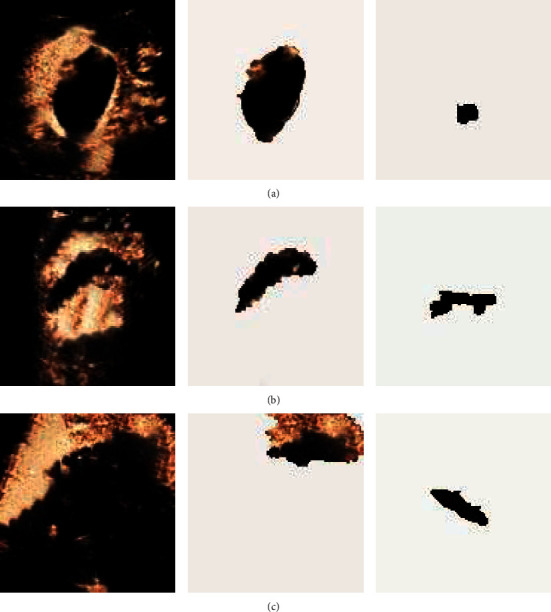
Recognition process of contrast-enhanced ultrasound images by GA. (a) Diagnosed as simple AC, with stones visible in the cyst, and the gallbladder wall was highly enhanced; (b) diagnosed as perforation of the gallbladder, the gallbladder was not dilated, there was a limited defect in the enhanced cyst wall, and fluid around the gallbladder; (c) diagnosed as acute gangrene gallbladder inflammation. There was localized thickening of the gallbladder wall, swelling, and fluid accumulation around the gallbladder.

Figure 6 is contrast-enhanced ultrasound image segmented by GA.  Figure 6(a) is the original gallbladder ultrasound image of the patient, Figure 6(b) represents the recognition of the gallbladder parenchyma contained in the patient image by the intelligent algorithm, and Figure 6(c) represents the lesion segmented by the intelligent algorithm.

**Figure 7:**
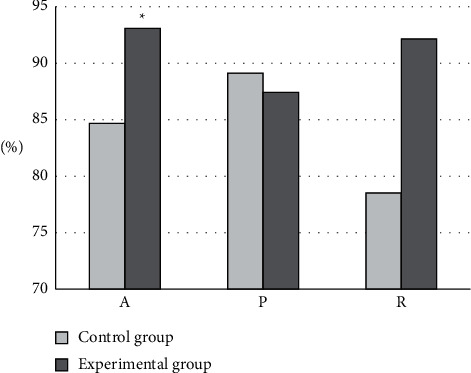
Diagnostic performance analysis. ^*∗*^Compared with the control group, *P* < 0.05.

In terms of the recognition effect of GA for contrast-enhanced ultrasound images, the diagnosis results of the GA were compared with the results by professional doctors, respectively. As shown in Figure 7, the average A, P, and *R* of professional doctors in the control group were 84.67%, 89.12%, and 78.53%, respectively, while the average A, P, and *R* of the intelligent algorithm were 93.08%, 87.42%, and 92.15%, respectively. Obviously, compared with the control group, the diagnostic accuracy of the experimental group was significantly different (*P* < 0.05).

**Figure 8:**
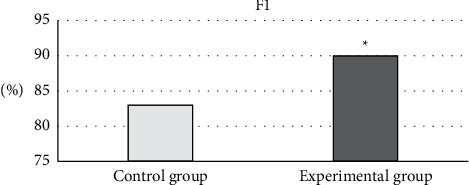
Comprehensive evaluation of P and (R) ^*∗*^Compared with the control group, *P* < 0.05.

As shown in Figure 8, when P and *R* conflicted, the F1 value of the professional doctors in the control group was 83%, and the F1 value of the algorithm in the experimental group was 90%. The intelligent recognition of the algorithm was significantly different from the F1 value of the doctor's diagnosis (*P* < 0.05).

### 3.4. The Optimal Interval Time to Perform LC after PTGD

**Figure 9:**
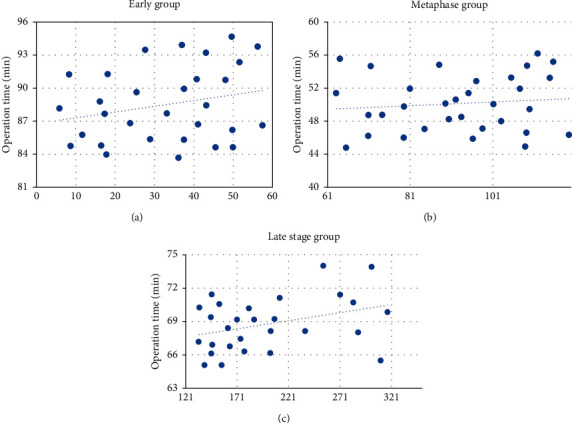
Average operation time: (a) the early group; (b) the middle group; (c) the late group.

The optimal interval time for performing LC after PTGD was analyzed. First, the operation time of LC at different intervals was recorded. As shown in Figure 9, the abscissa indicated the interval time, and the vertical axis indicated the operation time. The average operation time of 30 patients in the early group was 88.5 minutes; the average operation time of 32 patients in the middle group was 49.91 minutes; and the average operation time of 28 patients in the late group was 68.78 minutes.

**Figure 10:**
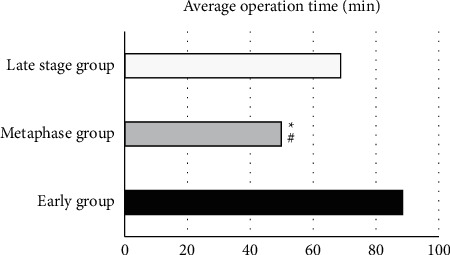
Comparison of the average operation time of the early group, the middle group, and the late group. ^*∗*^#Compared with the early and late group, respectively, *P* < 0.05.

Figure 10 is a comparison chart of the average operation time of the three groups. There was a significant difference compared to the middle group, *P* < 0.05.

**Figure 11:**
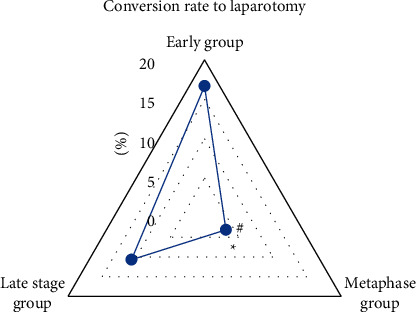
Comparison of the incidence of conversion to laparotomy in the early group, middle group, and late group. ^*∗*^# Compared with the early and late group, respectively, *P* < 0.05.

Then, the rate of transition from LC to laparotomy was analyzed. As shown in Figure 11, of 30 patients in the early group, 5 cases were converted to laparotomy, accounting for 16.67%; of 32 patients in the middle group, 1 case was converted to laparotomy, accounting for 3.13%; and of the 28 patients in the late group, 3 cases were converted to laparotomy, accounting for 10.71%. The middle group was significantly different from the early group and the late group, *P* < 0.05.

**Figure 12:**
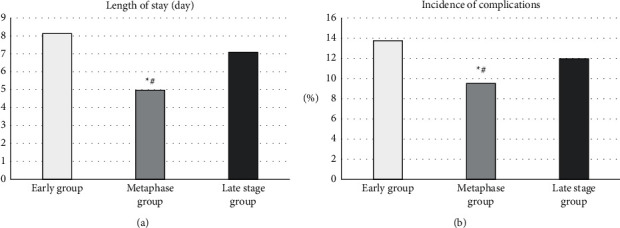
Comparison of the postoperative hospital stay and the incidence of complications in the early group, middle group, and late group: (a) the comparison chart of the length of hospital stay after the three groups; (b) the comparison chart of the incidence of complications in the three groups. ^*∗*^#Compared with the early and late group, respectively, *P* < 0.05.

Next, optimal interval to perform LC after PTGD was analyzed by the length of postoperative hospital stay. As shown in Figure 12(a), the average postoperative hospital stay of the early group was 8.13 days; the average postoperative hospital stay of the middle group was 4.97 days; and the average postoperative hospital stay of the late group was 7.09 days. Finally, the postoperative complications were analyzed. As shown in Figure 12(b), the complication rate in the early group was 13.75%; the complication rate in the middle group was 9.52%; and the complication rate in the late group was 11.96%. The middle group was significantly different from the early group and the late group, *P* < 0.05.

## 4. Discussion

The diagnosis of AC mainly relies on clinical history, physical examination, laboratory examination, and imaging examination [22]. Generally speaking, cholecystectomy should be performed within 72 hours after the onset of mild AC. Ultrasound is the preferred imaging examination method for suspected AC patients [23]. The gallbladder wall ultrasound imaging showing streaks, the degree of cholestasis, and gallbladder stone disease are all considered to be related to cholecystitis, and to intravenously inject ultrasound contrast agent can distinguish the diffuse defect of the gallbladder wall [24]. Contrast-enhanced ultrasound is a brand-new ultrasound examination technology developed in recent years. This technology has improved the routine ultrasound examination. After intravenous injection of ultrasound contrast agent [25], the ability to detect tissue blood perfusion is enhanced. The microvascular structure of normal and diseased tissue can be clearly displayed, thereby improving the accuracy of doctors' diagnosis. Contrast-enhanced ultrasound examination is simple, economical, efficient, and safe [26, 27], and it has been widely used in clinical practice. It is suitable for the situation where lesions are clearly shown on ordinary ultrasound image, but it is difficult to qualitatively diagnose. It is used for the diagnosis of various organ diseases and elevates the qualitative diagnosis ability of ultrasound, and the accuracy rate reaches 90% [28]. It is characterized by high safety, high cost performance, and efficient examination. The incidence of adverse reactions is less than 0.1%, and enhanced images and reports can be obtained immediately after examination, and there is no need to wait. GA was developed in the early 1960s and has now been widely used in research fields such as automatic control and image processing [28]. GA combined with contrast-enhanced ultrasound can facilitate diagnosis and the formulation of further surgical treatment plans, thereby reducing morbidity and mortality.

This study analyzed the effect of GA intelligent algorithm in identifying contrast-enhanced ultrasound, selected the most appropriate identification method to implement PTGD, and then got the most reasonable intermittent time for subsequent LC on this basis. It was found that contrast-enhanced ultrasound can more clearly distinguish the lesion than the routine ultrasound imaging. It can dynamically observe the filling range of the contrast agent in the gallbladder cavity so that the patency of the drainage tube can be monitored in real time, as well as its location in the gallbladder. In the control group, the average A, P, and *R* were 84.67%, 89.12%, and 78.53%, while in the experimental group, the average A, P, and *R* were, respectively, 93.08%, 87.42%, and 92.15%. Compared with the control group, the diagnostic accuracy of the experimental group was significantly different (*P* < 0.05), and the diagnostic accuracy of the intelligent algorithm was higher. The F1 value of the professional doctors in the control group was 83%; while the F1 value of the algorithm in the experimental group was 90%. Compared with the F1 value of the doctor's diagnosis, that of the intelligent algorithm was significantly different (*P* < 0.05), and the comprehensive effectiveness of the intelligent algorithm was higher. The average operation time of 30 patients in the early group was 88.5 minutes, the average operation time of 32 patients in the middle group was 49.91 minutes, and the average operation time of 28 patients in the late group was 68.78 minutes; the conversion rate of LC to laparotomy in the early group was 16.67%, in the middle group, it was 3.13%, and in the late group, it was 10.71%; the average postoperative hospital stay of the early group was 8.13 days, the average postoperative hospital stay of the middle group was 4.97 days, and the average postoperative hospital stay of the late group was 7.09 days; the incidence of complications in the early group was 13.75%, in the middle group, it was 9.52%, and in the late group, it was 11.96%. Above, the middle group was significantly different from the late group and the early group, *P* < 0.05. To conclude, intelligent algorithm-based ultrasound imaging could clearly identify the lesion sites and lesion characteristics of gallbladder and dynamically observe the filling range of contrast agent in the gallbladder cavity in real time. The safety and recovery speed of the implementation of LC within 2 to 4 months after surgery were better for patients.

## 5. Conclusion

In this study, the genetic intelligent algorithm was used to segment contrast-enhanced ultrasound images, and then the most reasonable intermittent duration of subsequent LC implementation was analyzed. It was found that contrast-enhanced ultrasound can more clearly distinguish the lesion of the gallbladder than the original ultrasound images. It can dynamically observe the filling range of the contrast agent in the gallbladder cavity. Additionally, the intelligent algorithm has higher diagnostic accuracy and comprehensive effectiveness. When it came to the optimal interval to perform LC after PTGD, LC performed within 2–4 months demonstrated better safety and recovery speed versus the early group and the late group. However, some limitations in the study should be noted. The sample size is small, which will reduce the power of the study. In the follow-up, an expanded sample size is necessary to strengthen the findings of the study. In conclusion, this study provides data and theoretical support for the clinical treatment of AC.

## Data Availability

The data used to support the findings of this study are available from the corresponding author upon request.
